# Diagnosis and Management of Acquired Hemophilia A: Case Reports and a Literature Review

**DOI:** 10.1155/2021/5554664

**Published:** 2021-09-14

**Authors:** Ikhwan Rinaldi, Findy Prasetyawaty, Siti Fazlines, Kevin Winston, Yusuf Aji Samudera Nurrobi, Jessica Leoni, Ilham Hidayat Restu Tulus Maha, Satrio Wicaksono, Abdillah Yasir Wicaksono, Averina Octaxena Aslani, Rizkania Ikhsani

**Affiliations:** ^1^Division of Hematology and Medical Oncology, Department of Internal Medicine, Cipto Mangunkusumo National General Hospital, Faculty of Medicine, Universitas Indonesia, Jakarta, Indonesia; ^2^Department of Internal Medicine, Cipto Mangunkusumo National General Hospital, Faculty of Medicine, Universitas Indonesia, Jakarta, Indonesia; ^3^Faculty of Medicine, Universitas Indonesia, Jakarta, Indonesia

## Abstract

**Background:**

Acquired hemophilia A (AHA) is a potentially life-threatening autoimmune hemostatic disorder where autoantibodies that disrupt the functions of factor VIII (FVIII) are present in the circulation. The early diagnosis of AHA is difficult since the symptoms of AHA differ from those of congenital hemophilia A. Furthermore, the management of AHA is also more complex due to the presence of autoantibodies against FVIII (FVIII inhibitors). Here, we present three case reports and conduct a literature review of AHA with the aim to increase awareness and knowledge regarding the diagnosis and treatment of AHA. *Case Presentations*. We present three patients diagnosed with AHA in these case reports. The first patient was a young female, while the second and third patients were middle-aged and elderly males, respectively. All patients presented with a chief complaint of bruises without hemarthrosis and a history of bleeding. Laboratory examinations of the patients revealed isolated prolonged aPTT, normal PT, and the presence of autoantibodies against factor VIII, which are characteristics of AHA. Patients were then treated with corticosteroids to reduce the titer level of autoantibodies and received factor VIII transfusion to stop bleeding.

**Conclusion:**

AHA can be suspected in patients presenting with symptoms of bruises without hemarthrosis and without the history of bleeding. Isolated aPTT elevation with normal PT should raise high suspicion of AHA. The presence of FVIII inhibitors can help to confirm the diagnosis of AHA. Treatment consists of factor VIII transfusion and corticosteroid therapy. Bypassing agents are recommended as an alternative to FVIII transfusion.

## 1. Introduction

Acquired hemophilia A (AHA) is a rare but potentially life-threatening autoimmune hemostatic disorder where autoantibodies against factor VIII are present in circulation due to a failure in immune tolerance [1, 2]. There are several fundamental differences between AHA and congenital factor VIII deficiency [1, 2]. The first is the presence of autoantibodies which neutralize the function of factor VIII in AHA. Second, the majority of AHA patients have cutaneous and intramuscular hemorrhage instead of hemarthrosis, which is commonly observed in congenital hemophilia A [2–5]. Additionally, the symptoms of AHA also vary across patients, with some having mild symptoms and others having major bleeding, which complicates diagnosis [2, 6]. Due to these differences and the lack of a past medical history of bleeding in many AHA patients, AHA is often not suspected or its diagnosis is difficult, which may delay the administration of treatment [2, 7]. Since the mortality rate of AHA is high, early diagnosis and management are important in reducing negative outcomes [3, 7–9].

The incidence rate of AHA has been estimated at around 1.2–1.48 cases per million people every year, with a mortality rate of around 20% [8–11]. Additionally, the incidence of AHA increases with age, with the elderly population being highly predisposed [3, 12, 13]. Furthermore, AHA may be associated with pregnancy, autoimmune diseases, and cancer [2, 3, 12, 13].

Here, we present case reports of AHA in three patients (a young female, middle-aged male, and elderly male) treated with either corticosteroids only or in combination with cyclophosphamide. By studying these cases and reviewing the recent guidelines, we humbly hope that this article may increase awareness and knowledge regarding the diagnosis and treatment of AHA.

## 2. Case Presentations

### 2.1. Case 1

An 18-year-old female presented on 7 April 2019 with a chief complaint of bruises on both legs in the past 2 weeks, which were painless but becoming larger over time. The patient also complained of an unhealed abdominal surgical wound 1 month after surgery due to a ruptured cyst in the right ovary.

Two months prior, the patient suffered a fall and was taken to a traditional massage therapist. However, she complained of small bruises after receiving the massage. Furthermore, the bruises increased in size over time. She was then treated with tranexamic acid at a clinic, with only slight improvement. She admitted that bruising had occurred easily throughout the previous year; however, she never consulted a physician because the bruises were relatively small and healed spontaneously. The patient denied having any history of liver dysfunction, blood clotting disorder, allergy, or any chronic disease. Similarly, the patient's mother also denied having any similar history of bleeding in the family.

On 7 April 2019, a physical examination of the patient was undertaken, showing stable vital signs and no other abnormalities. Laboratory investigation of blood taken on the same day revealed a normal complete blood count, normal PT of 10.7 s (normal value of 9.8–11.2 s), prolonged aPTT of 113.2 s (normal value of 31.0–47.0 s), elevated D-dimer of 610 *μ*g/L (normal value of <440 *μ*g/L), normal liver function, and normal kidney function. Additional laboratory results for factor VIII and factor IX levels were <1% (normal: 40–170%) and 63.5% (normal: 51–137%), respectively. Accordingly, factor VIII inhibitors were detected at 105.00 Bethesda units (BU) (normal: not detected). The patient was diagnosed with AHA on the basis of these clinical presentations and laboratory findings.

The next day, the patient underwent additional laboratory tests to exclude autoimmune diseases. These tests showed an ANA score of 1/320 with homogeneous patterns, while tests of anti-dsDNA, C3, and C4 were within normal limits. These results showed that autoimmune diseases were unlikely to be a precipitating factor of AHA.

**Figure 1:**
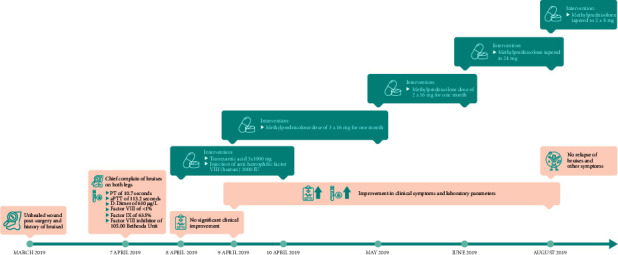
Timeline of events and interventions for case 1.

The patient was treated with both tranexamic acid (3 × 1000 mg) and injection of human factor VIII (2000 IU) for 2 days (8-9 April 2019); however, there was no significant clinical improvement. On 9 April 2019, it was decided to treat the patient with methylprednisolone (3 × 16 mg; equivalent to a prednisone dose of 1 mg/kg/day for 1 month). After 1 month of follow-up, her clinical presentation and laboratory parameters showed improvements. The corticosteroid treatment was then continued. In the second month, the methylprednisolone dose was reduced to 2 × 16 mg. Subsequently, in the third month, it was tapered to 24 mg (16–0–8 mg). In the fourth month of treatment, bruising no longer occurred. The methylprednisolone therapy was nevertheless continued with a dose of 2 × 8 mg, which was reduced by 4 mg weekly until the patient achieved steroid-free status. No relapse in terms of bruises or bleeding was observed after 6 months without steroid. Timeline of events for case 1 is shown in Figure 1.

### 2.2. Case 2

A 52-year-old male presented on 4 August 2019 with a chief complaint of bruises on all four limbs for the previous 3 weeks. The bruises were accompanied by swelling. According to the patient, the bruises first spontaneously appeared on his left foot before and later also spreading to other limbs.

The medical history was unremarkable, with the patient denying any history of trauma, prior liver dysfunction, chronic diseases, medications use, and blood clotting disorder. Additionally, the patient also denied having any similar history of bleeding in the family. Laboratory examinations on 5 August 2019 showed a prolonged aPTT of 87.8 s, decreased factor VIII level of 1.33%, and presence of factor VIII inhibitor at 106.80 BU (normal: not detected). Hence, the patient was diagnosed with AHA on the basis of the clinical manifestations and laboratory results.

On 5 August 2019, the patient received human factor VIII injections (3 × 2000 IU for 1 day), which then continued with a dose of 2 × 2000 IU for 2 days. Other treatments received by the patient were 3 × 1000 mg of tranexamic acid and 500 cc of a packed red cells transfusion. Furthermore, the patient was given methylprednisolone (1 × 125 mg IV for 5 days), with the dose subsequently reduced to 1 × 62.5 mg IV for 4 days.

On 13 August 2019, a second evaluation of factor VIII inhibitor and factor VIII levels was carried out, which showed improvements: 26.24 BU (normal: not detected) and 3.8% (normal: 40–170%). Meanwhile, the bruises on all four limbs were still present but had faded when compared with before, indicating a clinical improvement. On 14 August 2019, the dose of methylprednisolone was reduced to 3 × 16 mg PO for 2 weeks with the plan to reduce the dose further to 8 mg per week for the next 2 weeks. On the same day, tranexamic acid therapy was halted based on the improvements in bruises. The patient was then discharged from the hospital on 14 August 2019 and scheduled for an outpatient visit.

However, on 15 August 2019, the patient was admitted to the emergency unit after he accidentally fell while walking. Wide bruises appeared around the buttocks and groin, accompanied by pain. His laboratory tests revealed 7.1 g/dL of Hb and an aPTT of 47.8 s (normal: 31.0–47.0 seconds). As a result, the patient received supporting treatments consisting of packed red cells transfusion and human factor VIII injections (2 × 2000 IU for 3 days). Subsequently, recombinant factor VIII was used instead of factor VIII injections (2 × 1500 IU for 3 days), with the dose subsequently reduced to 1 × 1500 IU for 10 days and then 1 × 1500 IU every 2 days until the patient was discharged. The patient was also given methylprednisolone (32–32–16 mg) during the first 3 weeks of treatment, before continuing with 32–32–8 mg during the last week. Throughout the treatment period, the patient was also examined several times to exclude the possibility of having an autoimmune disease and cancer. The results for ANA and anti-dsDNA were negative. Furthermore, C3 and C4 were within normal limits.

**Figure 2:**
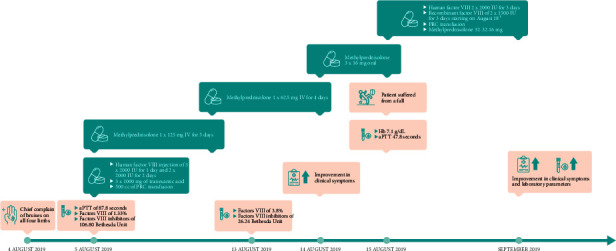
Timeline of events and interventions for case 2.

On 12 September 2019, there were significant improvements in clinical and coagulation parameters, with an aPTT of 31.5 s, factor VIII level of 33.2%, and an undetectable level of the factor VIII inhibitor. The patient was eventually discharged on 14 September 2019 and prescribed oral methylprednisolone (32–0–32 mg daily). The patient was advised to check his aPTT level every month, as well as factor VIII and factor VIII inhibitor levels 3 months later (January 2020), which were 8.1% and 3.16 BU, respectively. On the basis of these laboratory results, administration of cyclophosphamide tablets (2 mg/kg/day for 3 months) was considered according to a dose of 2 × 100 mg based on bodyweight of 96 kg. After the first 3 weeks of cyclophosphamide administration, factor VIII levels increased to 13.5%, whereas the inhibitor level was undetectable. Timeline of events for case 2 is shown in Figure 2.

### 2.3. Case 3

An 87-year-old male patient was admitted on 3 January 2019 for swelling and painless bruising in his left hand for the past month. The patient denied any history of trauma, prior liver dysfunction, or blood clotting disorder. Furthermore, the patient also denied having any similar history of bleeding in the family. The patient had comorbidities of chronic kidney disease (CKD) stage IIIB and prior cardiovascular disease (CVD).

One month prior to his admission, the patient had already been clinically diagnosed with AHA based on his clinical manifestations and laboratory findings (aPTT of 84.7 s) at a hospital in a different province. At that time, the patient was hospitalized and received a fresh frozen plasma transfusion, packed red cells PRC transfusion, and mycophenolate mofetil. The patient was then referred to a tertiary hospital for further evaluation.

A physical examination revealed the patient's blood pressure to be 159/90 mmHg, while other vital signs were stable. The initial complete blood count after a laboratory examination highlighted a hemoglobin level of 11.9 g/dL, white blood cell (WBC) count of 7.200/*μ*L, and platelet count of 305.000/*μ*L. The coagulation results showed an aPTT of 81.8 s (normal: 31.0–47.0 s). Further laboratory results showed a blood urea level of 107.9 mg/dL and serum creatinine level of 2 mg/dL.

Considering his clinical manifestation and prolonged aPTT, the patient was evaluated for factor VIII, factor IX, and factor VIII inhibitors, which were measured as <1% (normal: 40–170%), 109.5% (normal: 51–137%), and 10.56 BU (normal: undetected), respectively. Hence, the patient was diagnosed with AHA and chronic kidney disease.

**Figure 3:**
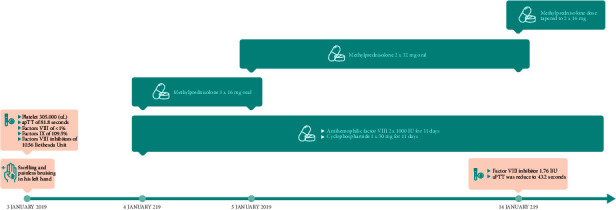
Timeline of events and interventions for case 3.

The patient was orally administered methylprednisolone (3 × 16 mg) on 4 January 2019, before reducing the dose to 2 × 32 mg the next day and 2 × 16 mg on 14 January 2019. The patient also received human factor VIII injections (2 × 1000 IU starting on 4 January 2019 for 11 days) and cyclophosphamide (1 × 50 mg). On 14 January 2019, the factor VIII inhibitor level was reduced to 1.76 BU, and the aPTT was reduced to 43.2 s. On 29 January, the patient had no complaints; thus, he was discharged and prescribed methylprednisolone (2 × 16 mg) for 2 months. On the day of discharge, the patient's aPTT was normal at 39.5 s (normal range: 31.0–47.0 s). Timeline of events for case 3 is shown in Figure 3.

## 3. Discussion

### 3.1. Diagnosis

The proper diagnosis of AHA is very important due to the associated mortality that results from severe bleeding. However, diagnosis is very challenging due to the rarity of the disease [2, 7, 9]. Another complicating factor is that AHA patients often have no history of bleeding or anticoagulant use [2, 5]. Furthermore, in contrast to hemarthroses which is common in congenital hemophilia A, bleeding in AHA predominantly occurs from the skin, muscles, and mucous membranes, making the symptoms nonspecific at best and confusing at worst [2, 4, 7, 14, 15].

Well-written clinical guidelines presented by Tiede et al., Kruse-Jarres et al., and Shetty et al. identified the first diagnostic pathway for suspected AHA as aPTT measurement [6, 16, 17]. The reason for this is that in AHA, there is often an isolated prolonged APTT without other abnormalities in coagulation, such as a prolonged PT or abnormal platelet count [5, 6, 16, 17]. However, an isolated prolonged aPTT can also be caused by deficiencies in other coagulation factors, such as FIX, FXI, and FXII [16]. Hence, additional diagnostic modalities are recommended to properly diagnose AHA.

An aPTT mixing test is helpful in differentiating causes of isolated prolonged aPTT. In a mixing test, the patient's plasma is mixed with an equivalent volume of normal plasma [18]. If a coagulation factor inhibitor is present, such as in AHA, the mixed plasma will still result in a prolonged aPTT due to the inhibition of coagulation factors. On the other hand, in a patient with only a coagulation factor deficiency (FVIII, FIX, FXI, and FXII), the mixing test would normalize the coagulation factors [2, 6, 16, 18].

The guidelines presented by Tiede et al. and Shetty et al. recommend conducting a mixing test after the aPTT test. By contrast, the guidelines by Kruse-Jarres et al. recommend that a FVIII activity concentration test be carried out in place of the mixing test (and after the aPTT test), which is only necessary if the FVIII test is unavailable [6, 16, 17]. Both Tiede et al. and Shetty et al. also recommended a FVIII test, but only after the mixing test is conducted. We agree with recommendation of Kruse-Jarres et al. that a mixing test may not be needed and can be skipped if an AHA diagnosis can already be confirmed by the low FVIII activity [6]. Another argument against the use of the mixing test is its lack of adequate standardization [17].

The measurement of the FVIII activity after aPTT test or mixing test is important as it can differentiate between the presence of AHA or lupus anticoagulants. A normal FVIII activity (≥50%) is highly indicative of lupus anticoagulants or other inhibitors such as FIX and FXI inhibitors, whereas a reduced FVIII activity level (<50%) instead indicates AHA [6, 16]. The presence of lupus anticoagulants can be excluded using the dilute Russell's viper venom time (dRVVT) assay [19]. The final step of AHA diagnosis is a Bethesda assay to detect and measure FVIII autoantibodies in Bethesda units (BU) [6, 16, 20].

### 3.2. Role of Hemostatic Agents

There are three focused objectives in AHA therapy [6, 12, 15]. The first objective is to control and prevent further bleeding, if present, to maintain the patient's vitals. The second objective is to eradicate factor VIII inhibitors in order to achieve complete remission. The third objective is to treat the underlying cause of the disease, if there is any.

For the first objective of bleeding control, hemostatic agents may be used. Currently, there are two types of hemostatic agents applied in AHA treatment: FVIII replacement therapy and bypassing agent (BPA) therapy [6, 12, 15, 21]. Human FVIII (hFVIII) replacement therapy has been a mainstay treatment for congenital hemophilia A. However, hFVIII is not effective in the presence of high anti-FVIII inhibitor titers, which may quickly neutralize the transfused hFVIII [22]. Nevertheless, when the antibody titer is <5 BU or BPA is not available, hFVIII replacement therapy can still be used as a first-line treatment, although the dose required is often high and the response may be unpredictable [2, 6, 7]. Indeed, a recent guideline by the World Federation of Hemophilia (WFH) in year 2020 also supported the use of FVIII replacement therapy for AHA patients with low-responding inhibitors (antibody titer < 5 BU) [23].

The issue of hFVIII inhibition such as in AHA patients with high antibody titer can be solved by using recombinant porcine-derived FVIII (rpFVIII), which should not be neutralized by the autoantibodies theoretically [24]. However, there have been several observations in the literature of some AHA patients having inhibitors which cross-react with rpFVIII, resulting in a lack of or reduction in response [24–26]. On the other hand, some patients may develop antiporcine FVIII antibody during therapy of rpFVIII [24]. The recommended dose of rpFVIII is 50–100 U/kg for patients with no detected antiporcine FVIII inhibitor [6]. If there is a detectable antiporcine FVIII inhibitor, the dose should be increased to 200 U/kg for severe bleeding; however, the dose remains 50–100 U/kg for less severe bleeding [6].

Several literatures have recommended BPAs, which activate blood clotting without a need for factor VIII, instead of using recombinant activated factor VII (rFVIIa) or plasma-derived activated prothrombin complex concentrate (aPCC) to control bleeding [6, 16, 17, 27–29]. Physiologically, FVIII activates FX in the coagulation cascade pathway. However, FX can also be activated both directly and indirectly by rFVIIa through tissue FVIIa [30]. Hence, a BPA such as rFVIIa can “bypass” the normal coagulation activation from FVIII, which is blocked by FVIII inhibitory antibodies. In the EACH2 study, rFVIIa and aPCC showed no significant difference in bleeding outcome [27]. However, it should be noted that there is a potential for rare thrombosis side effects from the use of BPAs [2, 17, 31, 32]. The recommended dosage of aPCC is 50–100 U/kg every 8−12 h, whereas that for rFVIIa is 90 *μ*g/kg every 2-3 h [16].

Generally, all AHA patients with bleeding should be administered hemostatic treatment. The choice of treatment depends mainly on treatment availability. BPAs should be chosen over replacement therapy if possible due to the advantage provided [2, 6, 16, 17]. The main limitations of BPAs are their expensive cost and lack of availability in several countries, where they are, thus, not the main treatment of choice, such as in Indonesia and other developing countries [27]. In these countries, factor VIII transfusion, therefore, remains the treatment of choice. A summary of the hemostatic therapies found in the literature is presented in Table 1.

### 3.3. Role of Tranexamic Acid and Desmopressin

Other hemostatic approaches, e.g., a combination of tranexamic acid with aPCC or rFVIIa, can help in maintaining clot stability [27, 33–35]. A study by Hvas et al. showed that a dose of 10 mg/kg is sufficient to achieve these effects [35].

Desmopressin which works by increasing FVIII plasma concentrations transiently through FVIII secretion from endothelial cells should only be used for mild bleeding and low titer inhibitors [17, 36, 37].

### 3.4. Role of Immunosuppressive Therapies

The elimination of factor VIII inhibitors can prevent future bleeding in AHA patients. The eradication of inhibitors using immunosuppressive therapy is recommended for all adults with AHA, despite some articles reporting that the presence of inhibitors can be spontaneously resolved to undetected levels [38, 39]. The consideration of this recommendation is based on the substantial mortality rate, morbidity rate, and cost of hemostatic treatment of acute bleeding [8]. However, due to risk of infections from immunosuppressive therapies which may cause mortality, extreme caution should be exercised [16].

There are currently three recommended immunosuppressive treatments: corticosteroid alone, corticosteroid combined with cyclophosphamide, or corticosteroid combined with rituximab [5, 6, 16]. The corticosteroid dose can either be 1 mg/kg prednisone daily or 40 mg dexamethasone daily for 3-4 weeks [6, 16]. The cyclophosphamide dose should be 1-2 mg/kg, whereas that for rituximab should be 375 mg/m^2^ IV weekly for 4 weeks [2, 6]. A summary of inhibitor eradication therapies found in the literature is presented in Table 2.

About 60–80% of patients achieved complete remission after a median of 5-6 weeks on immunosuppressive therapy [6]. Whether the administration of a corticosteroid combined with cyclophosphamide produces a better response than corticosteroid alone is still unclear. One study showed no statistically significant difference in median time to remission between both groups [9]. In contrast, the EACH2 study showed faster remission in patients receiving combination of steroids and cyclophosphamide when compared with steroids only [38]. Furthermore, a meta-analysis by Delgado et al. showed that the cyclophosphamide, with or without corticosteroids, was associated with better complete remission than corticosteroids only [40].

For rituximab, it is not shown to be superior than corticosteroids or cyclophosphamide in the EACH2 study; however, rituximab use was associated with longer time to remission [38]. Currently, many guidelines recommend rituximab as second-line therapy [2, 6, 16, 17, 28].

According to a prognostic study by Tiede et al., a low FVIII activity (<1 IU/dL) and >20 BU inhibitor titer were associated with a lower partial remission rate [39]. Furthermore, factors such as age, gender, and underlying disorder did not affect the partial remission rate [39]. The same study also observed that low FVIII activity (<1 IU/dL), >20 BU inhibitor titer, and age >74 years were predictors of poor overall survival [39]. Hence, it is recommended that corticosteroids be combined with rituximab or a cytotoxic agent such as cyclophosphamide as first-line therapy in patients with low FVIII activity and >20 BU inhibitor titer [16].

### 3.5. Role of Intravenous Immunoglobulin

The role of intravenous immunoglobulin (IVIG) as a first-line therapy exhibits no benefits, as shown by Delgado et al. in a review and meta-analysis [40]. Subsequent evidence also does not support the use of intravenous immunoglobulin for inhibitor eradication [9, 38]. For example, the EACH2 study showed no significant improvement from adding IVIG to immunosuppressive therapies [38]. Hence, IVIG is not recommended over other immunosuppressive therapies previously described [2, 6, 16, 17].

### 3.6. Lessons Learned

The first case report we presented demonstrates that AHA can occur in young females unrelated to pregnancy or autoimmune disease. A lack of pregnancy, therefore, does not necessarily exclude AHA diagnosis. Furthermore, we also reported two cases of idiopathic AHA. According to the cases above, an isolated subcutaneous hematoma without any bleeding history or other risk factors should warrant suspicion of AHA. Patients suspected of AHA should be screened for potential causes of AHA such as malignant diseases and autoimmune diseases.

Although it has been shown that the bleeding symptoms in AHA are usually severe, physicians should also be aware that mild bleeding can also occur in AHA [9, 16, 41]. A thorough laboratory examination must be performed to establish the diagnosis of AHA. Important laboratory examinations for AHA include the aPTT, mixing test, FVIII activity level, and FVIII inhibitor level [2, 6, 12, 16]. However, considering the presented cases, we argue that the mixing test may not be needed if an FVIII concentration test is available. Additional examinations for differential diagnoses, such as of lupus anticoagulants and VWF, may not be required in cases of low FVIII levels if there is a positive result in the Bethesda assay.

The treatment of AHA consists of hemostatic management and immunosuppressive therapy. The former constitutes BPAs as the first-line therapy, with FVIII replacement therapy used if BPA is not available. The latter is indicated as a combination therapy when high FVIII inhibitor titers and low FVIII levels are detected [6, 16].

These cases also demonstrate the poor correlation of FVIII inhibitor levels with bleeding severity. Therefore, physicians should make treatment decisions based not only on FVIII inhibitor levels but also the severity of bleeding symptoms.

### 3.7. Strengths and Limitations

The limitation of this case report is the lack of complete and uniform laboratory examinations in all patients. Additionally, the diagnostic and treatment pathways that followed differed from those in the recommended guidelines; however, due to these differences, the case reports offer new perspectives that can be beneficial for clinical practice, especially in developing countries where resources are limited, since all patients achieved clinical recoveries. Last, the follow-up of some patients after treatment was not long enough.

## 4. Conclusion

AHA is a rare hematologic disorder where factor VIII becomes inactive due to autoantibody formation. It is associated with increased morbidity and mortality. As it tends to occur in patients with an unknown history of bleeding disorders, early diagnosis is often difficult. Diagnosis of AHA is determined on the basis of the detection of low factor VIII levels in the presence of factor VIII inhibitors. The principles of AHA therapy involve controlling and preventing further bleeding, eradicating factor VIII inhibitors, and treating the underlying cause of the disease.

## Figures and Tables

**Table 1:** Summary of hemostatic therapies found in the literature.

Therapy	Authors		
	Tiede et al. [16]	Kruse-Jarres et al. [6]	Shetty et al. [17]
Human factor VIII	50–100 U/kg followed by tailored dosing If other options are unavailable	Dose not mentioned	Dose not mentioned

Recombinant porcine factor VIII (rpFVIII)	200 U/kg, followed by further doses to maintain levels >50%	If no antiporcine FVIII inhibitors present, 50–100 U/kg initially while monitoring FVIII activity every 2-3 h, with dose adjusted as required If antiporcine FVIII inhibitors are detected, 200 U/kg initially for severe bleeding or 50–100 U/kg for less severe bleeding	Not mentioned

Recombinant FVII activated (rFVIIa)	Bolus injections of 90 *μ*g/kg every 2-3 h until hemostasis is achieved	Dose of 70–90 *μ*g/kg every 2-3 h until hemostasis achieved	90 mcg/kg every 2 h until bleeding has been controlled

Activated prothrombin complex concentrate (APCC)	Bolus injections of 50−100 U/kg every 8−12 h, up to a maximum of 200 U/kg/day	Dose of 50–100 U/kg every 8–12 h not exceeding 200 U/kg/day	50–100 U/kg every 8–12 h with a maximum dose of 200 U/kg/day

**Table 2:** Summary of recommended inhibitor eradication therapies found in the literature.

Therapy	Authors		
	Tiede et al. [16]	Kruse-Jarres et al. [6]	Shetty et al. [17]
Corticosteroid	Prednisolone or prednisone 1 mg/kg/day orally for a maximum of 4–6 weeks	Prednisone 1 mg/kg/day orally daily or dexamethasone 40 mg orally daily	Prednisone 1 mg/kg/day orally daily

Rituximab	Dose of 375 mg/m^2^ for a maximum of four cycles	Dose of 375 mg/m^2^ IV weekly for 4 weeks or dose of 100 mg weekly for 4 weeks	Dose of 375 mg/m^2^ IV weekly for 4 weeks

Cytotoxic agents	Cyclophosphamide 1.5–2 mg/kg/day orally for a maximum of 6 weeks or mycophenolic acid (MMF) 1 g/day for 1 week, followed by 2 g/day	Cyclophosphamide 1-2 mg/kg orally daily or cyclophosphamide ∼5 mg/kg IV for 3-4 weeks	Cyclophosphamide 1-2 mg/kg daily

## Data Availability

No data were used to support this study.

## References

[1] Ma A. D., Carrizosa D. (2006). Acquired factor VIII inhibitors: pathophysiology and treatment. *Hematology ASH Education Program*.

[2] Charlebois J., Rivard G.-É., St-Louis J. (2018). Management of acquired hemophilia A: review of current evidence. *Transfusion and Apheresis Science*.

[3] Knoebl P., Marco P., Baudo F. (2012). Demographic and clinical data in acquired hemophilia A: results from the European Acquired Haemophilia Registry (EACH2). *Journal of Thrombosis and Haemostasis*.

[4] Janbain M., Leissinger C. A., Kruse-Jarres R. (2015). Acquired hemophilia A: emerging treatment options. *Journal of Blood Medicine*.

[5] Haider M. Z., Anwer F. (2020). *Acquired Hemophilia*.

[6] Kruse-Jarres R., Kempton C. L., Baudo F. (2017). Acquired hemophilia A: updated review of evidence and treatment guidance. *American Journal of Hematology*.

[7] Collins W., W Collins P., Chalmers E. (2013 Sep). Diagnosis and management of acquired coagulation inhibitors: a guideline from UKHCDO. *British Journal of Haematology*.

[8] Bitting R. L., Bent S., Li Y., Kohlwes J. (2009). The prognosis and treatment of acquired hemophilia: a systematic review and meta-analysis. *Blood Coagulation and Fibrinolysis*.

[9] Collins P. W., Hirsch S., Baglin T. P. (2007). Acquired hemophilia A in the United Kingdom: a 2-year national surveillance study by the United Kingdom haemophilia centre doctors’ organisation. *Blood*.

[10] Collins P., Macartney N., Davies R., Lees S., Giddings J., Majer R. (2004). A population based, unselected, consecutive cohort of patients with acquired haemophilia A. *British Journal of Haematology*.

[11] Tay L., Duncan E., Singhal D. (2009). Twelve years of experience of acquired hemophilia A: trials and tribulations in South Australia. *Seminars in Thrombosis and Hemostasis*.

[12] Tiede A., Werwitzke S., Scharf R. E. (2014). Laboratory diagnosis of acquired hemophilia A: limitations, consequences, and challenges. *Seminars in Thrombosis and Hemostasis*.

[13] Huang S.-Y., Tsay W., Lin S.-Y., Hsu S.-C., Hung M.-H., Shen M.-C. (2015). A study of 65 patients with acquired hemophilia A in Taiwan. *Journal of the Formosan Medical Association*.

[14] Franchini M., Gandini G., Paolantonio T. D., Mariani G. (2005). Acquired hemophilia A: A concise review. *American Society of Hematology*.

[15] Windyga J., Baran B., Odnoczko E. (2019). Treatment guidelines for acquired hemophilia A. *Ginekologia Polska*.

[16] Tiede A., Collins P., Knoebl P. (2020). International recommendations on the diagnosis and treatment of acquired hemophilia A. *Haematologica*.

[17] Shetty S., Bhave M., Ghosh K. (2011). Acquired hemophilia a: diagnosis, aetiology, clinical spectrum and treatment options. *Autoimmunity Reviews*.

[18] Kershaw G., Orellana D. (2013). Mixing tests: diagnostic aides in the investigation of prolonged prothrombin times and activated partial thromboplastin times. *Seminars in Thrombosis and Hemostasis*.

[19] Jacquot C., Wool G. D., Kogan S. C. (2019). Dilute Russell viper venom time interpretation and clinical correlation: a two‐year retrospective institutional review. *The International Journal of Literary Humanities*.

[20] Duncan E., Collecutt M., Street A. (2013). Nijmegen-Bethesda assay to measure factor VIII inhibitors. *Haemostasis*.

[21] Collins P., Baudo F., Huth-Kühne A. (2010). Consensus recommendations for the diagnosis and treatment of acquired hemophilia A. *BMC Research Notes*.

[22] Nemes L., Pitlik E. (2000). New protocol for immune tolerance induction in acquired hemophilia. *Haematologica*.

[23] Srivastava A., Santagostino E., Dougall A. (2020). WFH guidelines for the management of hemophilia. *Haemophilia Off J World Federation of Hemophilia*.

[24] Kruse-Jarres R., St-Louis J., Greist A. (2015). Efficacy and safety of OBI-1, an antihaemophilic factor VIII (recombinant), porcine sequence, in subjects with acquired haemophilia A. *Haemophilia*.

[25] Türkantoz H., Königs C., Knöbl P., Klamroth R., Holstein K., Huth‐Kühne A. (2020). Cross-reacting inhibitors against recombinant porcine factor VIII in acquired hemophilia A: Data from the GTH-AH 01/2010 Study. *Journal of Thrombosis and Haemostasis*.

[26] Tarantino M. D., Cuker A., Hardesty B., Roberts J. C., Sholzberg M. (2017). Recombinant porcine sequence factor VIII (rpFVIII) for acquired haemophilia A: practical clinical experience of its use in seven patients. *Haemophilia*.

[27] Baudo F., Collins P., Huth-Kühne A. (2012). Management of bleeding in acquired hemophilia A: results from the European Acquired Haemophilia (EACH2) Registry. *Blood*.

[28] Franchini M., Castaman G., Coppola A. (2015). Acquired inhibitors of clotting factors: AICE recommendations for diagnosis and management. *Blood Transfus*.

[29] Ilkhchoui Y., Koshkin E., Windsor J. J., Petersen T. R., Charles M., Pack J. D. (2013). Perioperative management of acquired hemophilia a: a case report and review of literature. *Anesthesiology and Pain Medicine*.

[30] Hoffman M., Dargaud Y. (2012). Mechanisms and monitoring of bypassing agent therapy. *Journal of Thrombosis and Haemostasis*.

[31] Borg J.-Y., Négrier C., Durieu I., Dolimier E., Masquelier A.-M., Lévesque H. (2015). FEIBA in the treatment of acquired haemophilia A: results from the prospective multicentre French ’FEIBA dans l’hémophilie A acquise’ (FEIBHAC) registry. *Haemophilia*.

[32] Bykov K., Bohn R., Ewenstein B., Seeger J., Avorn J., Bateman B. (2017). Use of bypassing agents and risk of thromboembolic events in patients with haemophilia and inhibitors. *Thrombosis & Haemostasis*.

[33] Tran H. T. T., Sørensen B., Rea C. J., Bjørnsen S., Ueland T., Pripp A. H. (2014). Tranexamic acid as adjunct therapy to bypassing agents in haemophilia A patients with inhibitors. *Haemophilia*.

[34] Holmström M., Tran H. T. T., Holme P. A. (2012). Combined treatment with APCC (FEIBA) and tranexamic acid in patients with haemophilia A with inhibitors and in patients with acquired haemophilia A - a two-centre experience. *Haemophilia*.

[35] Hvas A.-M., Sørensen H. T., Norengaard L., Christiansen K., Ingerslev J., Sørensen B. (2007). Tranexamic acid combined with recombinant factor VIII increases clot resistance to accelerated fibrinolysis in severe hemophilia A. *Journal of Thrombosis and Haemostasis*.

[36] Rosenberg J. B., Greengard J. S., Montgomery R. R. (2000 Dec). Genetic induction of a releasable pool of factor VIII in human endothelial cells. *Arteriosclerosis, Thrombosis, and Vascular Biology*.

[37] Franchini M., Lippi G. (2011). The use of desmopressin in acquired haemophilia A: a systematic review. *Blood Transfusion*.

[38] Collins P., Baudo F., Knoebl P. (2012). Immunosuppression for acquired hemophilia a: results from the european acquired haemophilia registry (EACH2). *Blood*.

[39] Tiede A., Klamroth R., Scharf R. E. (2015). Prognostic factors for remission of and survival in acquired hemophilia A (AHA): Results from the GTH-AH 01/2010 study. *Blood*.

[40] Delgado J., Jimenez-Yuste V., Hernandez-Navarro F., Villar A. (2003). Acquired haemophilia: review and meta-analysis focused on therapy and prognostic factors. *British Journal of Haematology*.

[41] Yousphi A. S., Bakhtiar A., Cheema M. A., Nasim S., Ullah W. Acquired Hemophilia A: a rare but potentially fatal bleeding disorder. *Cureus*.

